# Supplementary figures and images for: Cerebral Apolipoprotein D Exits the Brain and Accumulates in Peripheral Tissues
Source: Int J Mol Sci. 2021 Apr 16;22(8):4118. doi: 10.3390/ijms22084118 (PMC8073497; doi:10.3390/ijms22084118)

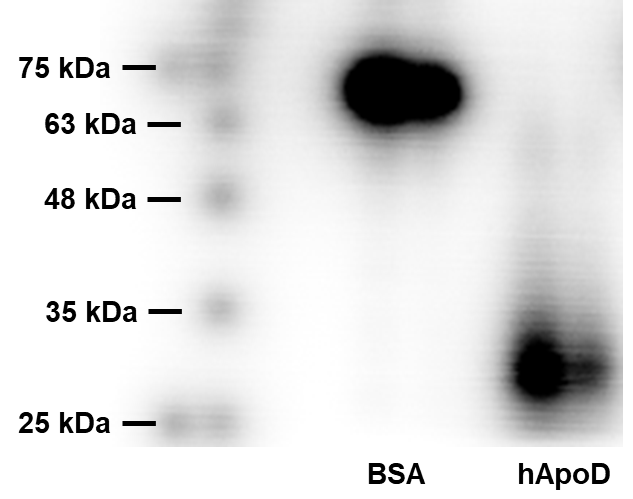

Supplement: Supplementary file 1 [file ijms-22-04118-s001.zip › Sup. Fig 1.png]

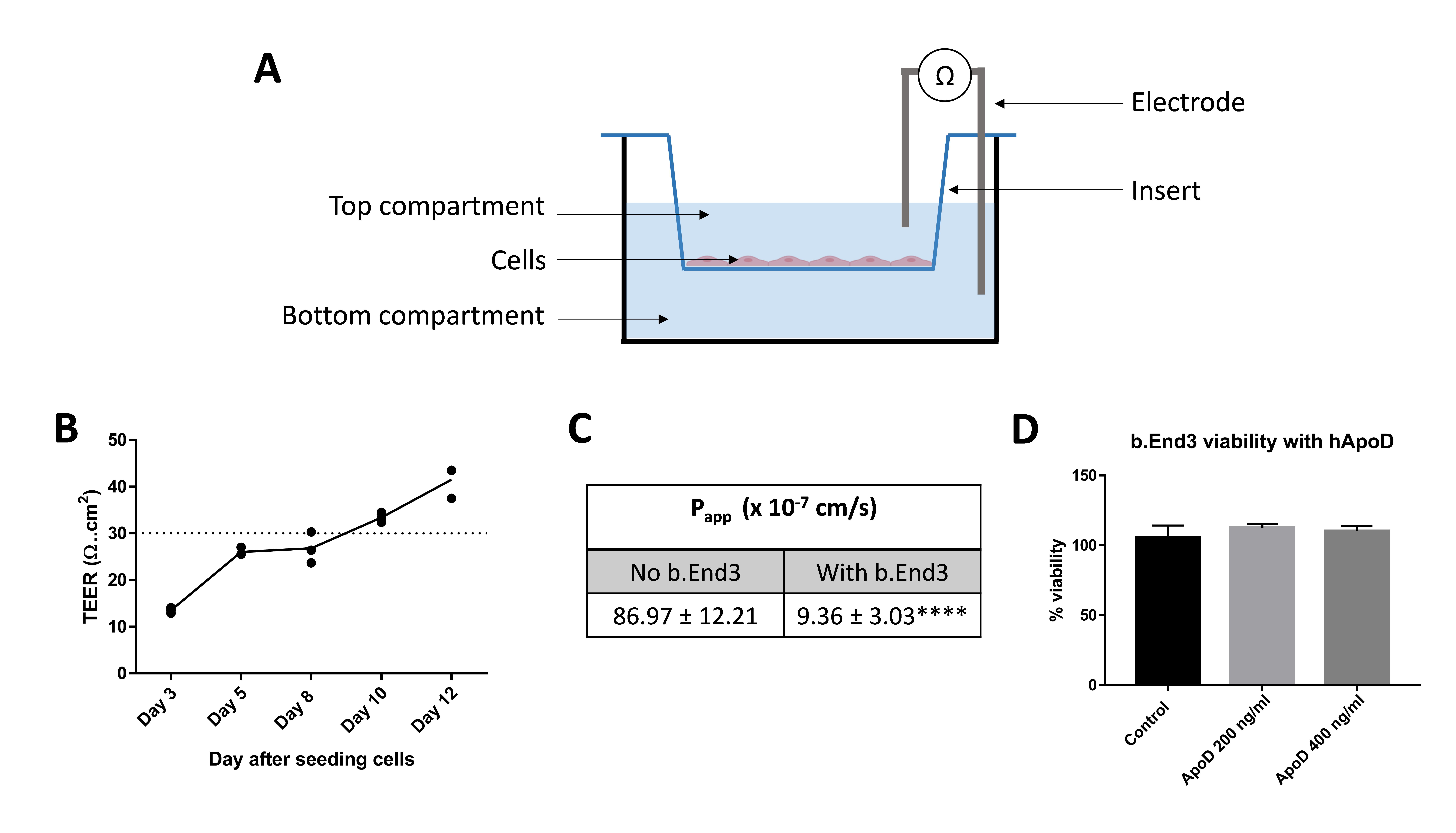

Supplement: Supplementary file 1 [file ijms-22-04118-s001.zip › Sup. Fig 2.jpg]

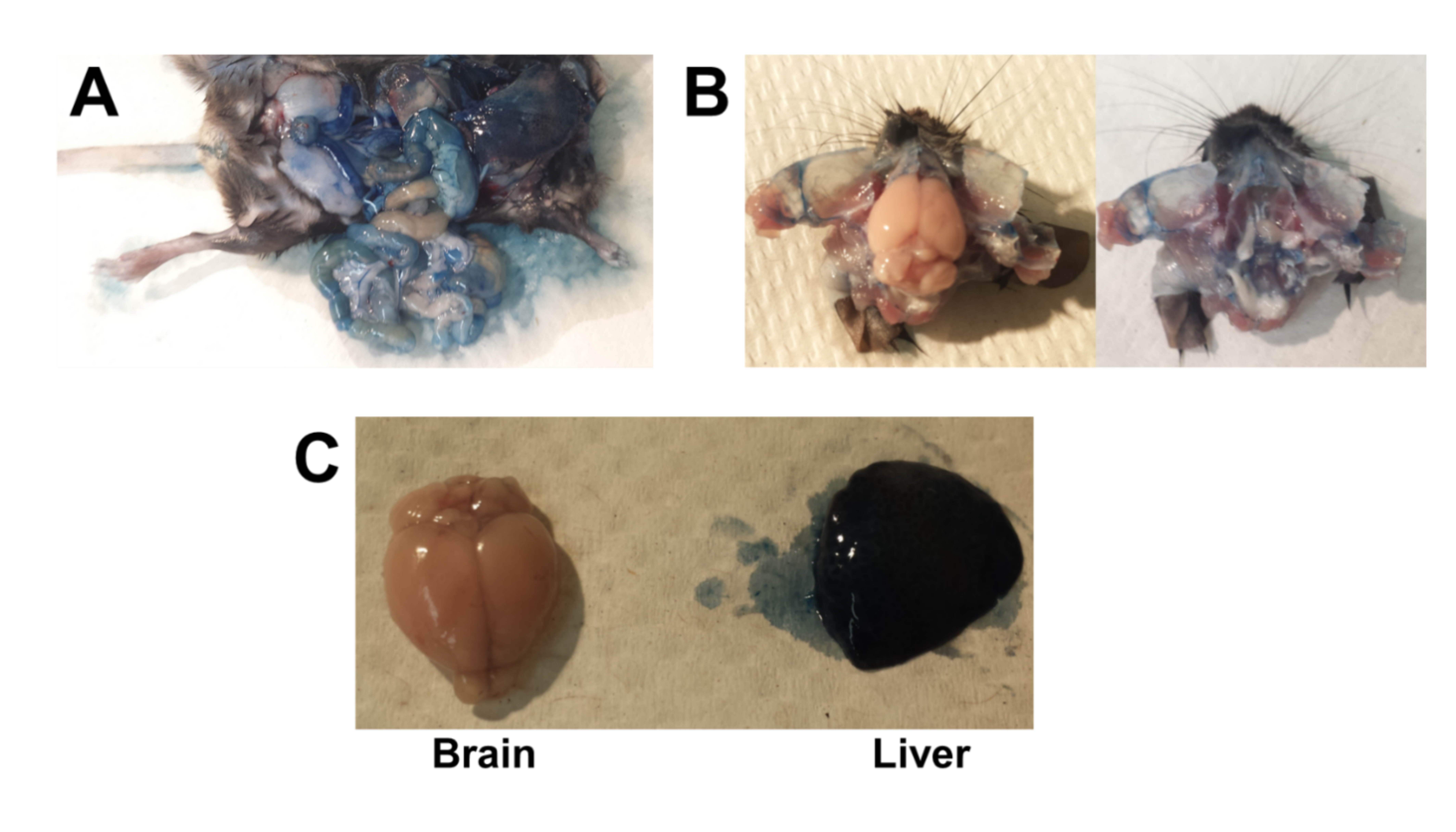

Supplement: Supplementary file 1 [file ijms-22-04118-s001.zip › Sup. Fig 3.tif]
